# Broad Range Eubacterial Polymerase Chain Reaction of Cerebrospinal Fluid Reduces the Time to Exclusion of and Costs Associated with Ventriculostomy-Related Infection in Hemorrhagic Stroke

**DOI:** 10.1007/s12028-023-01888-x

**Published:** 2023-12-12

**Authors:** Elisabeth Pietrzko, Stefan Bögli, Katja Frick, Sabeth Ebner-Dietler, Crescenzo Capone, Frank Imkamp, Hendrik Koliwer-Brandl, Nicolas Müller, Emanuela Keller, Giovanna Brandi

**Affiliations:** 1 Neurocritical Care Unit, Institute of Intensive Care, University Hospital of Zürich, Rämistrasse 100, 8091 Zürich, Switzerland; 2 Department of Neurology, Clinical Neuroscience Center Zürich, University Hospital Zürich, Frauenklinikstrasse 26, 8091 Zürich, Switzerland; 3 Institute of Medical Microbiology, University of Zürich, Gloriastrasse 28/30, 8006 Zürich, Switzerland; 4 Department of Infectious Diseases and Hospital Epidemiology, University Hospital Zürich, Rämistrasse 100, 8091 Zürich, Switzerland; 5 Department of Neurosurgery, University Hospital Zürich, Frauenklinikstrasse 10, 8091 Zürich, Switzerland

**Keywords:** Eubacterial polymerase chain reaction, Ventriculostomy-related infection, Health care-associated ventriculitis, Cerebrospinal fluid, Hemorrhagic stroke

## Abstract

**Background:**

Patients with hemorrhagic stroke and an external ventricular drain in situ are at risk for ventriculostomy-related-infections (VRI). Because of the contamination of the cerebrospinal fluid (CSF) with blood and the high frequency of false negative CSF culture, the diagnosis of VRI remains challenging. This study investigated the introduction of CSF broad range eubacterial polymerase chain reaction (ePCR) and its effect on frequency and duration of antibiotic therapy for VRI, neurocritical care unit (NCCU) length of stay, related costs, and outcome.

**Methods:**

Between 2020 and 2022, we prospectively included 193 patients admitted to the NCCU of the University Hospital of Zürich with hemorrhagic stroke and an external ventricular drain for more than 48 h. Patient characteristics, serum inflammatory markers, white blood cell count in CSF, use and duration of antibiotic treatment for VRI, microbiological findings (CSF cultures and ePCR tests), and NCCU length of stay were compared in patients with no infection, noncerebral infection, suspected VRI, and confirmed VRI. Data of patients with suspected VRI of this cohort were compared with a retrospective cohort of patients with suspected VRI treated at our NCCU before the introduction of CSF ePCR testing (2013–2019).

**Results:**

Out of 193 patients, 12 (6%) were diagnosed with a confirmed VRI, 66 (34%) with suspected VRI, 90 (47%) with a noncerebral infection, and 25 (13%) had no infection at all. Compared with the retrospective cohort of patients, the use of CSF ePCR resulted in a reduction of patients treated for suspected VRI for the whole duration of 14 days (from 51 to 11%). Furthermore, compared with the retrospective group of patients with suspected VRI (*n* = 67), after the introduction of CSF ePCR, patients with suspected VRI had shorter antibiotic treatment duration of almost 10 days and, hence, lower related costs with comparable outcome at 3 months.

**Conclusions:**

The use of CSF ePCR to identify VRI resulted in shorter antibiotic treatment duration without changing the outcome, as compared with a retrospective cohort of patients with suspected VRI.

**Supplementary Information:**

The online version contains supplementary material available at 10.1007/s12028-023-01888-x.

## Introduction

Ventriculostomy-related infections (VRI) are frequent in patients with hemorrhagic stroke and are associated with increased morbidity and mortality [1–4]. Risk factors for VRI include duration of catheterization, presence of subarachnoid hemorrhage (especially in patients with intraventricular hemorrhage), penetrating or open traumatic brain injury, basilar skull fractures with cerebrospinal fluid (CSF) leak, and neurosurgical procedures [5].

Various factors hinder diagnosing VRI. Currently, no standardized definition of VRI exists [6]. Thus, the reported prevalence of VRI varies widely (from 0 to 22%) with an average incidence of 8% [4, 7]. After a hemorrhagic stroke, CSF pleocytosis, glucose, and protein levels should be evaluated cautiously in patients with suspected VRI, due to the contamination with blood [2]. Similarly, fever, headache, and meningeal signs—described criteria for VRI—might be associated to the underlying disease further than to VRI [8, 9]. Furthermore, following prior antibiotic therapy, up to 20% of the CSF cultures might return as a false negative result [3].

The use of broad range eubacterial polymerase chain reaction (ePCR) for detection of bacterial agents in CSF is promising, however, its use is still not widely established. Bacterial DNA can be detected by amplification (polymerase chain reaction [PCR]) and subsequent sequence analysis of the *16S* rRNA gene [10, 11]. Compared with the classical microbiology assays, ePCR results return sooner, they are less prone to false negative results in patients previously treated with antibiotic therapy, and they might reveal novel etiologic microorganisms [6].

In neurosurgical patients, due to postsurgical changes, invasive devices, and prior antibiotic exposure [12–14]_,_ the diagnosis of VRI remains challenging. Moreover, in patients with hemorrhagic stroke, CSF contamination with blood makes the interpretation of the laboratory values even more difficult [15]. Previous studies conducted in patient populations with hemorrhagic stroke and VRI did not investigate the role of CSF ePCR [16–18]. Additionally, studies that investigated the role of the CSF ePCR in patients with VRI did not refer to this specific patients’ population [19–22].

Thus, the objective of this study was to determine whether the introduction of routine CSF ePCR in patients with hemorrhagic stroke and suspected VRI had resulted in a reduction of the frequency of “confirmed” VRI, in a reduction of the length of stay (LOS) at the intensive care unit (ICU), in a comparable or better outcome, and in a reduction of the use of antibiotic medication for VRI treatment in terms of duration and costs compared with a patient cohort treated at our ICU prior to the introduction of ePCR.

## Methods

The study was approved by the local ethics committee (Kantonale Ethikkommission Zürich, BASEC-Nr2021-00631) and was in accordance with the ethical standards laid down in the 2013 Declaration of Helsinki for research involving human study participants.

### Study Population, Inclusion, and Exclusion Criteria

All patients admitted between January 1st, 2020, and November 30th, 2022, to the neurocritical care unit (NCCU) of the University Hospital Zürich were prospectively screened for eligibility. Inclusion criteria were (1) age (> 18 years); (2) insertion of an external ventricular drain (EVD) at the Department of Neurosurgery, University Hospital Zürich; (3) EVD in situ for more than 48 h. Exclusion criteria was patient’s written or documented oral refusal to have his or her data analyzed for research projects.

### EVD Management and VRI Screening

At our institution, EVDs are inserted by a neurosurgical consultant in the operating room or at the NCCU under sterile conditions. Silver impregnated lines (Silverline, Spiegelberg, Germany) are used. Prophylactic antibiotic therapy with cefuroxime is administered as a single shot. CSF samples are routinely collected twice weekly and additionally in case of a suspected VRI. CSF is extracted from the EVD under sterile conditions. The first 2 ml of CSF are discarded. The next 3 ml of CSF are used for laboratory analyses. Because of the large proportion of patients with intraventricular blood, measurements of CSF glucose, lactate, and protein are not helpful and thus not routinely performed. The white blood cell count (WBC) within CSF is corrected for the number of erythrocytes (red blood cell count [RBC]) within the sample, according to the formula: corrected WBC = reported CSF-WBC – ([WBC in peripheral blood × RBC in CSF] / [RBC in peripheral blood]).

### VRI Diagnostic Criteria

The diagnostic criteria used in our institution are based on the recommendations of the Infectious Diseases society of America (IDSA) [8]. The exact clinical and laboratory criteria used are described below. The main difference between the recommendations of the IDSA and our local protocol is in the use of ePCR in addition to CSF culture to confirm the presence of or to exclude a VRI. A VRI is suspected and treated with antibiotics in case of at least two of the following conditions starting two or more days after EVD-insertion:

- Clinical signs of VRI (fever, defined as a temperature of ≥ 38.3 °C measured in-ear/bladder or > 38 °C measured within the brain tissue, meningeal irritability, and/or unclear neurological deterioration not better explained by an alternative cause, e.g., increasing intracranial pressure, progression of hemorrhage, cerebral vasospasm, etc.). Similarly, the IDSA recommends that new headache, nausea, lethargy, and or change in mental status, as well as fever, in the absence of another clear source of infection, could be suggestive of VRI [8].
- WBC in CSF > 500/µl (set as a local cutoff both based on the local experience and previous research [23]). Because of the frequent occurrence of intraventricular blood, the IDSA considers CSF cell count potentially not be a reliable indicator for the presence of infection in patients with VRI [8].
- Elevated systemic inflammatory parameters (C-reactive protein [CRP] > 5 mg/l, procalcitonin [PCT] > 0.1 μg/l, and/or WBC > 9.6 G/l, measured daily) not better explained by an alternative cause (excluded by performing blood, bronchial and urinary cultures, plain chest x-rays, and physical examination). Similarly, the IDSA elevated serum PCT potentially useful in differentiating between CSF abnormalities due to surgery or intracranial hemorrhage from those due to bacterial infection [8].

Patients with suspected VRI are treated initially empirically with intravenous antibiotics such as vancomycin and meropenem, as recommended by the IDSA [8]. In case of negative CSF Gram staining (available within 24 h) despite VRI suspicion, a CSF ePCR is performed and a CSF culture is inoculated. Before January 2020 (i.e., before ePCR was added to the diagnostic work-up), antibiotic therapy was continued until a negative CSF culture was confirmed (up to 14 days after inoculation, following the IDSA guidelines that recommend an inoculation of at least 10 days). After January 2020, we decided to stop antibiotic therapy when a negative CSF ePCR was available. In patients with CSF contamination, defined as a positive CSF culture and/or ePCR result with skin pathogens (e.g., *Staphylococcus epidermidis*) without clinical suspicion of VRI therapy is also stopped.

For the analysis, the study population was divided in four groups:

1. Confirmed VRI: a confirmed VRI is defined by a positive CSF culture or positive ePCR with concurrent clinical suspicion of VRI. Therapy is always adapted to the detected bacteria and their antibiotic resistance spectrum.
2. Suspected VRI: initial VRI suspicion based on at least two criteria listed above (a–c), but negative CSF culture and CSF ePCR results.
3. No infection: no or only one criterion listed above and no signs of noncerebral infection.
4. Noncerebral infection: clinical signs of other sites of infection (e.g., pneumonia, urinary tract infection or catheter-associated bloodstream infection) with elevated systemic inflammatory parameters (CRP > 5 mg/l, PCT > 0.1 μg/l, and/or WBC > 9.6 G/l, measured daily) as well as negative CSF culture and ePCR.

The two groups “no infection” and “noncerebral infection” were analyzed to investigate and compare the role of the systemic inflammatory parameters in patients with no CSF infection and patients with confirmed or suspected VRI.

### Broad Range ePCR

Eubacterial polymerase chain reaction for the detection of bacterial pathogens was performed according to a semiautomated workflow described recently [11]. In brief, DNA was extracted from samples using the QIASymphony platform (Qiagen, Hilden, Germany), followed by an automated PCR set-up. PCRs were run on a Lightcycler 480-II (Roche, Basel, Switzerland). Amplicons of positive samples (i.e., a 500-bp fragment of the bacterial *16S* rRNA gene) were subjected to Sanger-sequencing. Identification of pathogens was done by analyzing sequencing data using the *16S* rDNA SmartGene Integrated Database Network System (IDNS) custom platform following the identification guidelines published previously [24]. Chromatograms containing overlapping peaks, indicative for the presence of two or more pathogens, were additionally analyzed using the software Ripseq (Pathogenomix, Santa Cruz, CA, USA) to potentially achieve further differentiation.

### Data Collection

Data were obtained by prospective evaluation of the electronic health records for patients’ characteristics, clinical and radiological records, and laboratory values as well as microbiological findings. Patients’ characteristics (age, sex, initial Glasgow coma scale [GCS], main diagnosis, and presence of intraventricular hemorrhage on first brain computed tomography scan (CT)), antibiotic treatment of any kind and specific for VRI, number of days of antibiotic treatment for VRI, length of ICU stay (NCCU-LOS) (expressed in days) were collected. Furthermore, microbiological findings (CSF culture and CSF ePCR results) were listed, as well as CSF-WBC. The systemic inflammatory parameters, including CRP, PCT, and WBC were collected. For the statistical analysis, only the peak values of CSF-WBC and of the systemic inflammatory parameters during the ICU stay with the EVD in situ were considered.

Outcome was assessed by two NCCU physicians and presented using the Glasgow Outcome Scale Extended (GOSE) [25] at 3 months routine follow up consultation (which includes a neurological examination, as well as description of current occupation including the percentage of working capability).

Data from this prospective cohort were compared with a previously described retrospective cohort with suspected VRI treated at our NCCU between 2013 and 2019 before the introduction of CSF ePCR testing [23]. Diagnostic criteria for VRI for the retrospective cohort are available in the supplementary materials (list, Suppl. Materials). In the retrospective cohort, a VRI was considered “confirmed” in case of a positive CSF culture. In patients with suspected and confirmed VRI, the antibiotic therapy for VRI was than given for at least 14 days, as recommended, independently from the results of the CSF cultures to not miss false negative cultures, above all following previous antibiotics administration [8]. In particular, patients’ characteristics, systemic inflammatory parameters, antibiotic treatment, duration of antibiotic treatment for VRI, NCCU-LOS, and GOSE at 3 months were compared.

In this study, we compared duration of antibiotic treatments for VRI, their related costs, and NCCU-LOS in patients with suspected VRI prospectively and retrospectively (*n* = 67) included. For the evaluation of antibiotic therapy related costs, we assumed an average patient weight of 70 kg and multiplied daily costs of vancomycin and meropenem by the number of days the therapy was given to each patient.

### Statistical Analysis

Statistical analysis was performed using SPSS version 28. Descriptive statistics are reported as counts/percentages, mean ± standard deviation, or as median including the interquartile range as appropriate. All continuous data were tested for normality using Shapiro–Wilk’s test. Categorical or ordinal variables are compared with Pearson’s *χ*^2^ or Fisher’s exact test, continuous variables using Student’s *t*-test or Mann–Whitney *U*-test for parametric and nonparametric data, respectively, when appropriate. For comparison between multiple groups, a Kruskal–Wallis test or Pearson’s *χ*^2^ including Bonferroni correction was used.

## Results

Overall, 193 patients fulfilled the inclusion criteria of the study. Of them, 104 patients (54%) suffered from an aneurysmal subarachnoid hemorrhage and 60 patients (31%) from an intracerebral hemorrhage, the remaining 29 (15%) from other diagnoses (e.g., nonaneurysmal spontaneous subarachnoid hemorrhage).

Twelve (6%) patients presented with a confirmed VRI, 66 (34%) with a suspected VRI, 25 (13%) had no infection at all, and 90 (47%) a noncerebral infection. In patients with confirmed VRI, the following microorganisms were detected: *Streptococcus anginosus* (*n* = 3), coagulase negative staphylococci (*n* = 3), *Serratia marcescens* (*n* = 1), *Enterobacter cloacae* (*n* = 1), *Klebsiella pneumoniae* (*n* = 1), *Klebsiella aerogenes* (*n* = 1), *Pseudomonas* spp. (*n* = 1), and *Fusibacterium* spp. (*n* = 1), as shown in Table 6. In this group, CSF cultures were positive in seven patients, and CSF ePCR was positive and CSF culture was negative in five patients, as shown in Table 1. One patient had a positive CSF culture, which was interpreted as contamination.

**Table 1:** Demographics and characteristics of the patients from the prospective cohort

Demographic	Confirmed VRI (*n* = 12)	suspected VRI (*n* = 66)	*p* value (MWU-test/*χ*^2^)
Age (years)	58 ± 13.9	56 ± 14.5	0.740
Initial GCS ≤ 8, *n* (%)	3 (25.0)	24 (36.4)	0.527
Diagnosis (%)			0.016
SAH	3 (25.0)	42 (63.6)	< 0.05
ICH	4 (33.3)	16 (24.2)	n.s
Other	5 (41.7)	8 (12.1)	< 0.05
IVH, *n* (%)	6 (50.0)	47 (71.2)	
NCCU-LOS (days)	12 (14.25–29.5)	18 (15–30)	0.647
Hospital-LOS (days)	44 (26–54.5)	27.5 (21–41.25)	0.031

Data are expressed as mean ± standard deviation, or median and interquartile range, as appropriate. Percentages are shown in parentheses

GCS, Glasgow Coma Scale, ICH: intracranial hemorrhage, IVH, intraventricular hemorrhage, LOS, length of stay, MWU, Mann–Whitney-U, NCCU, neurocritical care unit, n.s., not significant, SAH, subarachnoid hemorrhage, VRI, ventriculostomy-related infection

Comparisons among all four groups regarding age, initial GCS, main diagnosis, NCCU-LOS, and hospital LOS are available in the supplementary materials (Table 1, Suppl. Materials).

Age, initial GCS, and main diagnosis were comparable between patients with suspected and confirmed VRI (Table 1). Additionally, patients with confirmed VRI had longer hospital LOS than patients with suspected VRI (*p* = 0.031).

The systemic inflammatory parameters and CSF-WBC in the different groups are presented in the supplementary materials (Table 2, Suppl materials). In particular, CRP was similar in patients with no infection and confirmed VRI. PCT was similar among all groups. CSF-WBC was higher in patients with a confirmed VRI. The systemic inflammatory parameters and CSF-WBC did not differ between patients with confirmed and suspected VRI (Table 2).

**Table 2:** Systemic inflammatory parameters of the patients from the prospective cohort

Parameter	Confirmed VRI (*n* = 12)	suspected VRI (*n* = 66)	*p* value (MWU-test/*χ*^2^)
Plasma CRP (mg/dl)	92 (53–278.3)	120 (66.3–189.3)	0.857
Plasma PCT (yg/l)	0.08 (0.07–0.15)	0.14 (0.07–0.4)	0.129
Plasma WBC (4–10 × 10^9^/l)	13.5 (8.1–5.3)	11.2 (9.14–13.94)	0.722
CSF WBC (4–10 × 10^9^/l)	348 (209–1013)	280 (88–665)	0.312

Data are expressed as mean ± standard deviation, or median and interquartile range, as appropriate. On the right side of the table are presented the comparisons of the systemic inflammatory parameters between the different groups

CRP, protein C -reactive, CSF, cerebrospinal fluid, MWU, Mann–Whitney-U, PCT, procalcitonin, VRI, ventriculostomy-related infection, WBC, white blood cell count

Patients with suspected VRI from our prospective cohort (*n* = 66) were compared with those from a retrospective cohort of patients with suspected VRI (*n* = 67) treated at our NCCU before the introduction of the CSF ePCR testing (Table 3). In the retrospective cohort (2013–2019), a total of 263 patients with hemorrhagic stroke, an EVD in situ for more than 48 h, and at least one CSF sample were treated at our NCCU as previously described [19]. Of those, 81 patients were treated for VRI and 14 had positive CSF culture. Considering the two groups of patients with suspected VRI (prospectively and retrospectively included), they were comparable regarding mean age, initial GCS, duration of EVD in situ, and administration of antibiotic therapy before the diagnosis of suspected VRI. Patients with suspected VRI after the introduction of the CSF ePCR had shorter durations of antibiotic treatment for VRI than the retrospective group (5.5 [3–9] days vs. 14 [3–14], *p* < 0.001). The results of the CSF ePCR were available on average 5 days after sampling.

**Table 3:** Comparisons between patients with suspected VRI retrospectively (*n* = 67) and prospectively (*n* = 66) included before and after the introduction of the broad range eubacterial polymerase chain reaction of cerebrospinal fluid

Demographic	Retrospective group (*n* = 67)	Prospective group (*n* = 67)	*p* value
Age (years)	58 ± 12.0	56 ± 14.5	0.35
Initial GCS ≤ 8	29 (43.3)	24 (36.4)	0.48
Diagnosis			0.001
SAH	59 (88.1)	42 (63.6)	< 0.05
ICH	8 (11.9)	16 (24.2)	n.s
Other	0 (0.0)	8 (12.1)	< 0.05
IVH	38 (56.7)	47 (71.2)	0.104
EVD days in situ (days)	7 (5–7)	7 (5–10)	0.982
Prior antibiotic treatment (yes)	47 (70.1)	41 (62.1)	0.461
Duration antibiotic treatment for sVRI	14 (3–14)	5.5 (3, 9)	0.001
Price of AB treatment/patient (CHF)	27,332.2 (5856.9–7332.3)	10,737.65 (5856.9–17,570.5)	0.001
NCCU length of stay (d)	20 (14–28)	18 (15–30)	0.746
Hospital length of stay (d)	28 (22–34)	28 (21–41)	0.709

Data are expressed as mean ± standard deviation, or median and interquartile range, as appropriate. Percentages are shown in parentheses

AB, antibiotic treatment, CHF, Swiss francs, EVD, external ventricular drainage, GCS, Glasgow Coma Scale, ICH, intracerebral hemorrhage, IVH, intraventricular hemorrhage, NCCU, neurocritical care unit, n.s., not significant, SAH, subarachnoid hemorrhage, sVRI, suspected ventriculostomy-related infection, VRI, ventriculostomy-related infection

NCCU LOS and hospital LOS were comparable in patients with suspected VRI in the retrospective and in the prospective cohort (Table 3).

Outcome between prospectively and retrospectively included patients with suspected VRI as assessed with GOSE at 3 months was comparable (Table 4). In the prospective cohort, outcomes between patients with suspected and confirmed VRI are presented in Table 5. Patients with confirmed VRI had more often an unfavorable outcome (GOSE 1–4) than patients with suspected VRI (*p* = 0.016).

**Table 4:** Outcome comparison between patients with suspected VRI retrospectively (*n* = 67) and prospectively (*n* = 66) included before and after the introduction of the broad range eubacterial polymerase chain reaction of cerebrospinal fluid

GOSE	Retrospective group (*n* = 67)	Prospective group (*n* = 66)	*p* value
Unfavorable (1–4)	32 (47.8)	40 (60.1)	0.881
1	9 (16.7)	10 (15.2)	
2	4 (7.4)	4 (6.1)	
3	10 (18.5)	13 (19.7)	
4	9 (16.7)	13 (19.7)	
5	12 (22.2)	17 (25.8)	
6	9 (16.7)	4 (6.1)	
7	1 (1.9)	3 (4.5)	
8	0 (0.0)	2 (3.0)	

Outcome was assessed with the Glasgow Outcome Scale extended (GOSE) at 3 months. Percentages are shown in parentheses

VRI, ventriculostomy-related infection

**Table 5:** Outcome comparison between patients with suspected (*n* = 66) and confirmed VRI (*n* = 12) from the prospective cohort

GOSE	sVRI (*n* = 66)	confirmed VRI (*n* = 12)	*p* value
Unfavorable (1–4)	40 (60.1)	12 (100.0)	0.016
1	10 (15.2)	2 (16.7)	
2	4 (6.1)	2 (16.7)	
3	13 (19.7)	3 (25.0)	
4	13 (19.7)	5 (41.7)	
5	17 (25.8)	0 (0.0)	
6	4 (6.1)	0 (0.0)	
7	3 (4.5)	0 (0.0)	
8	2 (3.0)	0 (0.0)	

Outcome was assessed with the Glasgow Outcome Scale extended (GOSE) at 3 months. Percentages are shown in parentheses

sVRI, suspected ventriculostomy-related infection, VRI, ventriculostomy-related infection

After the introduction of the CSF ePCR, estimated costs of antibiotic therapy were significantly lower (Table 3). Alongside the reduction of treatment duration and costs, the introduction of ePCR resulted in a significant reduction in the number of patients treated with antibiotics for the whole duration of 14 days after suspicion for VRI as compared with the retrospective cohort (from 51 to 11%, *p* < 0.001). After discontinuing the antibiotic therapy in patients with suspected VRI, none of them had a new clinical or laboratory suspicion of a VRI (Table 6).

**Table 6:** Patients from the prospective study with “confirmed” ventriculostomy-related infection (VRI) (*n* = 12)

Patient ID	Microorganism	CSF culture positive	CSF ePCR positive
1	*Fusibacterium* spp.		x
2	*Pseudomonas* spp.		x
3	*Streptococcus anginosus*	x	
4	*Serratia marcescens*	x	
5	*Klebsiella pneumoniae*	x	
6	*Enterobacter cloacae*	x	
7	*Streptococcus intermedius*		x
8	*Streptococcus intermedius*		x
9	*Staphylococcus coagulase negative*	x	
10	*Staphylococcus coagulase negative*	x	
11	*Klebsiella aerogenes*		x
12	*Staphylococcus coagulase negative*	x	

The microorganisms found in cerebrospinal fluid (CSF) of patients with “confirmed” VRI are listed below. The diagnosis was confirmed by positive CSF culture and positive broad range eubacterial polymerase chain reaction (ePCR) of cerebrospinal fluid

ID, identification, spp., Plural, several species

## Discussion

The present study was conducted to evaluate whether the introduction of CSF ePCR in patients with hemorrhagic stroke and suspected VRI at our NCCU improved the diagnostic pathway of VRI, reduced overtreatment, and hence, reduced use of antibiotics, and lead to shorter ICU stay, without impacting the patient’s outcome.

In our cohort, the introduction of CSF ePCR reduced the number of patients treated for suspected VRI for the complete duration of 14 days from 51 to 11% due to an earlier exclusion of VRI and consequently decreased the duration of antibiotic treatment by almost 10 days. This led to a decrease in costs, without worsening the neurological outcome. These results suggest that CSF ePCR is a useful tool and, in comparison to the gold standard of CSF culture, aids earlier exclusion of VRI in patients with hemorrhagic stroke.

Considering the financial impact of these results, we compared the estimated costs for the antibiotic treatment due to suspected VRI comparing the prospective cohort with our retrospective cohort treated at the same NCCU before and after the introduction of CSF ePCR. Although this calculation is only approximate, and the relevant reduction of costs for antibiotic therapy for VRI after the introduction of the ePCR is only estimated, the reduction of the duration of antibiotic therapy for VRI after the introduction of the ePCR is relevant, both in terms of reducing health care costs and avoiding the rise of multiresistant microorganisms [26, 27]. NCCU LOS and hospital LOS in patients with suspected VRI did not differ between the retrospective and the prospective cohort. Although surprising, this result implies that other factors aside from suspected VRIs are the cause of extended hospitalizations (e.g., cerebral vasospasms, rebleeding etc.).

## Limitations

This study has some limitations. Firstly, this is the experience of a single center, limiting the generalizability of the results. A possible role of the CSF ePCR for the diagnosis of VRI should be further evaluated and validated. Secondly, currently there is no “gold standard” to define VRI in patients with hemorrhagic stroke and CSF contamination with blood. Although our criteria follow the guidelines set by the IDSA, it is likely that some suspected VRIs were either aseptic meningitis or low inoculum infections leading to wrong grouping. However, considering that after discontinuation of the antibiotic for VRI in the case of negative ePCR there were no new clinical and laboratory suspicions of VRI, we are convinced that the use of the CSF ePCR in these patients helped VRI exclusion, and, hence, reduced overtreatment.

Thirdly, the small sample size of patients with confirmed VRI does not allow further analysis to better characterize this group. We cannot exclude that we treated patients with false positive CSF ePCR. Another limitation of the study is that the control group was not matched and only few baseline characteristics were compared between groups. On the other hand, we included all consecutively admitted patients with hemorrhagic stroke and EVD in situ and thus reduces the risk of selection bias. Finally, the limitations of the ePCR should be considered: ePCR has primarily been validated for sterile materials [10]. ePCR usually only provides meaningful results in monobacterial infections, which is the case in most patients with VRI [28, 29]. Although ePCR can detect a wide spectrum of bacterial DNA through *16S* RNA PCR sequencing, the sensitivity of ePCR is lower than species-specific amplification methods [11, 30]. If a specific infection is highly suspected, additional species-specific detection methods might be beneficial [10, 11].

## Conclusions

The introduction of CSF ePCR led to shorter durations of antibiotic therapy due to earlier exclusion of VRI and correspondingly lower estimated costs without worsening the outcome of the patients, as compared with patients treated at our unit before the introduction of the CSF ePCR.

### Supplementary Information

Below is the link to the electronic supplementary material.

Supplementary file1 (DOCX 18 kb)

## References

[1] Lozier AP, Sciacca RR, Romagnoli MF, Connolly ES (2008). Ventriculostomy-related infections: a critical review of the literature. Neurosurgery.

[2] Mayhall CG, Archer NH, Lamb VA, Spadora AC, Baggett JW, Ward JD (1984). Ventriculostomy-related infections: a prospective epidemiologic study. N Engl J Med.

[3] Habib OB, Srihawan C, Salazar L, Hasbun R (2017). Prognostic impact of health care-associated meningitis in adults with intracranial hemorrhage. World Neurosurg.

[4] Martin RM, Zimmermann LL, Huynh M, Polage CR (2018). Diagnostik approach to health care- and device-associated central nervous system infections. J Clin Microbiol.

[5] Sam JE, Lim CL, Sharda P, Wahab NA (2018). The organisms and factors affecting outcomes of external ventricular drainage catheter-related ventriculitis: a penang experience. Asian J Neurosurg..

[6] Karvouniaris M, Brotis A, Tsiakos K, Palli E, Koulenti D (2022). Current perspectives on the diagnosis and management of healthcare-associated ventriculitis and meningitis. Infect Drug Resist.

[7] Dey M, Jaffe J, Stadnik A, Awad IA (2012). External ventricular drainage for intraventricular hemorrhage. Curr Neurol Neurosci Rep.

[8] Tunkel AR, Hasbun R, Bhimraj A, Byers K, Kaplan SL, Scheld WM, van de Beek D, Bleck TP, Garton HJL, Zunt JR (2017). Infectious Diseases Society of America’s clinical practice for healthcare-associated ventriculitis and meningitis. Clin Infect Dis.

[9] Centers for Disease Control and Prevention/National Healthcare Safety Network. Surveillance definitions for specific types of infections. NHSN; 2023.

[10] Rampini SK, Bloemberg GV, Keller PM, Büchler AC, Dollenmaier G, Speck RF, Böttger EC (2011). Broad-range 16 S rRNA gene polymerase chain reaction for diagnosis of culture-negative bacterial infections. Clin Infect Dis.

[11] Wagner K, Springer B, Pires VP, Keller PM (2019). High-troughput screening of bacterial pathogens in clinical specimens using 16 S rDNA qPCR and fragment analysis. Diagnosis Microbiol Infect Dis.

[12] Hasbun R (2021). Healthcare-associated ventriculitis: current and emerging diagnostic and treatment strategies. Expert Rev Anti Infect Ther.

[13] Muttaiyah S, Ritchie S, Upton A, Roberts S (2008). Clinical parameters do not predict infection in patients with external ventricular drains: a retrospective observational study of daily cerebrospinal fluid analysis. J Med Microbiol.

[14] Meredith FT, Phillips HK, Reller LB (1997). Clinical utility of broth cultures of cerebrospinal fluid from patients at risk for shunt infections. J Clin Microbiol.

[15] Hoogmoed J, van de Beek D, Coert BA, Horn J, Vandertop WP, Verbaan D (2017). Clinical and laboratory characteristics for the diagnosis of bacterial ventriculitis after aneurysmal subarachnoidal hemorrhage. Neurocrit Care.

[16] Montes K, Jenkinson H, Habib OB, Esquenazi Y, Hasbun R (2019). Corrected white blood cell count, cell index, and validation of a clinical model for the diagnosis of health care-associated ventriculitis and meningitis in adults with intracranial hemorrhage. Clin Neurol Neurosurg.

[17] Murthy SB, Moradiya Y, Shah J, Hanley DF, Ziai WC (2016). Incidence, predictors, and outcomes of ventriculostomy-associated infections in spontaneous intracerebral hemorrhage. Neurocrit Care.

[18] Lenski M, Huge V, Schmutzer M, Ueberschaer M, Briegel J, Tonn JC, Schichor C, Thon N (2019). Inflammatory markers in serum and cerebrospinal fluid for early detection of external ventricular drain–associated ventriculitis in patients with subarachnoid hemorrhage. J Neurosurg Anesthesiol.

[19] Banks JT, Bharara S, Tubbs RS (2005). Polymerase chain reaction for the rapid detection of cerebrospinal fluid shunt or ventriculostomy infections. Neurosurgery.

[20] Rath PM, Schoch B, Adamzik M, Steinmann E, Buer J, Steinmann J (2014). Value of multiplex PCR using cerebrospinal fluid for diagnosis of ventriculostomy-related meningitis in neurosurgery patients. Infection.

[21] Dabrowski P, Jurkiewicz J, Czernicki Z, Koszewski W, Jasielski P (2017). Polymerase chain reaction based detection of bacterial 16S rRNA gene in the cerebrospinal fluid in the diagnosis of bacterial central nervous system infection in the course of external cerebrospinal fluid drainage. Comparison with standard diagnostics currently used in clinical practice. Neurol Neurochir Pol.

[22] Lenski Gordon CL, Tokarz R, Briese T, Lipkin WI, Jain K, Whittier S, Shah J (2015). Evaluation of a multiplex polymerase chain reaction for early diagnosis of ventriculostomy-related infections. J Neurosurg.

[23] Bögli SY, Wang SS, Pietrzko E (2022). Plasma inflammatory markers and ventriculostomy-related infection in patients with hemorrhagic stroke: a retrospective and descriptive study. Front Neurol.

[24] Bosshard PP, Abels S, Zbinden R, Böttger EC, Altwegg M (2003). Ribosomal DNA sequencing for identification of aerobic gram-positive rods in the clinical laboratory (an 18-month evaluation). J Clin Microbiol.

[25] Desai A, Lollis SS, Missios S (2009). How long should cerebrospinal fluid cultures be held to detect shunt infections?. J Neurosurg Pediatrics.

[26] Styers D, Sheehan DJ, Hogan P, Sahm DF (2006). Laboratory-based surveillance of current antimicrobial resistance patterns and trends among *Staphylococcus aureus*: 2005 status in the United States. Ann Clin Microbiol Antimicrob.

[27] Musher DM, Dowell ME (2002). Emergence of macrolide resistance during treatment of pneumococcal pneumonia. N Engl J Med.

[28] Dorresteijn KR, Jellema K, van de Beek D, Brouwer MCJN (2019). Factors and measures predicting external CSF drain-associated ventriculitis: a review and meta-analysis. Neurology.

[29] Widén J, Eriksson B-M, Ronne-Engström E, Enblad P, Westman G (2017). Ventriculostomy-related infections in subarachnoid hemorrhage patients—a retrospective study of incidence, etiology, and antimicrobial therapy. Acta Neurochir.

[30] Morel AS, Dubourg G, Prudent E, Edouard S, Gouriet F, Casalta JP, Fenollar F, Fournier PE, Drancourt M, Raoult D (2015). Complementarity between targeted real-time specific PCR and conventional broad-range 16 S rDNA PCR in the syndrome-driven diagnosis of infectious diseases. Comparative study. Eur J Clin Microbiol Infect Dis.

