# Correction: Broad Range Eubacterial Polymerase Chain Reaction of Cerebrospinal Fluid Reduces the Time to Exclusion of and Costs Associated with Ventriculostomy-Related Infection in Hemorrhagic Stroke

**DOI:** 10.1007/s12028-023-01926-8

**Published:** 2024-02-05

**Authors:** Elisabeth Pietrzko, Stefan Bögli, Katja Frick, Sabeth Ebner-Dietler, Crescenzo Capone, Frank Imkamp, Hendrik Koliwer-Brandl, Nicolas Müller, Emanuela Keller, Giovanna Brandi

**Affiliations:** 1 Neurocritical Care Unit, Institute of Intensive Care, University Hospital of Zürich, Rämistrasse 100, 8091 Zürich, Switzerland; 2 Department of Neurology, Clinical Neuroscience Center Zürich, University Hospital Zürich, Frauenklinikstrasse 26, 8091 Zürich, Switzerland; 3 Institute of Medical Microbiology, University of Zürich, Gloriastrasse 28/30, 8006 Zürich, Switzerland; 4 Department of Infectious Diseases and Hospital Epidemiology, University Hospital Zürich, Rämistrasse 100, 8091 Zürich, Switzerland; 5 Department of Neurosurgery, University Hospital Zürich, Frauenklinikstrasse 10, 8091 Zürich, Switzerland

**Correction to: Neurocritical Care** 10.1007/s12028-023-01888-x

The original article has been corrected. The addresess of affiliations have been updated.

