# Supplementary material for: Broad Range Eubacterial Polymerase Chain Reaction of Cerebrospinal Fluid Reduces the Time to Exclusion of and Costs Associated with Ventriculostomy-Related Infection in Hemorrhagic Stroke
Source: Neurocrit Care. 2023 Dec 12;40(3):1109–16. doi: 10.1007/s12028-023-01888-x (PMC11147905; doi:10.1007/s12028-023-01888-x)
Supplement: Supplementary file 1 — Supplementary file1 (DOCX 18 kb) [file 12028_2023_1888_MOESM1_ESM.docx]

**Supplementary materials**

Supplementary table 1. Patients’ demographics and characteristics from the prospective cohort. Data are expressed as mean ± standard deviation, or median and interquartile range, as appropriate. Percentages are shown in brackets. *GCS:* Glasgow Coma Scale; *ICH:* intracranial hemorrhage; *IVH*: intraventricular hemorrhage; *LOS:* length of stay; *NCCU:* neurocritical care unit; *VRI:* ventriculostomy-related infection; *SAH:* subarachnoid hemorrhage.

|  | no infection (0) | non-cerebral infection (1) | VRI (2) | suspected VRI (3) | p-value | 0 vs 1 | 0 vs 2 | 0 vs 3 | 1 vs 2 | 1 vs 3 | 2 vs 3 |
| --- | --- | --- | --- | --- | --- | --- | --- | --- | --- | --- | --- |
|  | n=25 | n=90 | n=12 | n=66 | KW-test/Chi2 |  |  |  |  |  |  |
| Age [years] | 56±16.5 | 62±16.1 | 58±13.9 | 56±14.5 | 0.081 |  |  |  |  |  |  |
| Initial GCS ≤ 8 (%) | 8 (32.0) | 44 (48.9) | 3 (25.0) | 24 (36.4) | 0.172 |  |  |  |  |  |  |
| Diagnosis (%) |  |  |  |  | 0.052 |  |  |  |  |  |  |
| SAH | 10 (40.0) | 49 (54.4) | 3 (25.0) | 42 (63.6) |  |  |  |  |  |  |  |
| ICH | 12 (48.0) | 28 (31.1) | 4 (33.3) | 16 (24.2) |  |  |  |  |  |  |  |
| other | 3 (12.0) | 13 (14.4) | 5 (41.7) | 8 (12.1) |  |  |  |  |  |  |  |
| IVH (%) | 22 (88.0) | 63 (70.0) | 6 (50.0) | 47 (71.2) | 0.104 |  |  |  |  |  |  |
| NCCU- LOS [days] | 6 [3, 12.5] | 14 [8, 22.25] | 12 [14.25, 29.5] | 18 [15, 30] | <0.001 | <0.001 | <0.001 | <0.001 | 0.422 | <0.001 | 0.697 |
| Hospital- LOS [days] | 14 [4.5, 18.5] | 23 [10.5, 31] | 44 [26, 54.5] | 27.5 [21, 41.25] | <0.001 | 0.050 | <0.001 | <0.001 | 0.001 | 0.001 | 0.549 |

Supplementary table 2. Systemic inflammatory parameters of the patients from the prospective cohort. Data are expressed as mean ± standard deviation, or median and interquartile range, as appropriate. Percentages are shown in brackets. *CRP*: protein C -reactive, *CSF*: cerebrospinal fluid, *PCT:* Procalcitonin, *WBC*: White blood cell count. On the right side of the table are presented the comparisons of the systemic inflammatory parameters between the different groups.

|  | no infection (0) | non-cerebral infection (1) | Confirmed  VRI  (2) | suspected VRI  (3) | p-value | 0 vs. 1 | 0 vs. 2 | 0 vs. 3 | 1 vs. 2 | 1 vs. 3 | 2 vs. 3 |
| --- | --- | --- | --- | --- | --- | --- | --- | --- | --- | --- | --- |
|  | n=25 | n=90 | n=12 | n=66 | KW-test/Chi2 |  |  |  |  |  |  |
| Plasma CRP [mg/dl] | 53 [33.5, 73.5] | 140 [80.8, 199.5] | 92 [53, 278.3] | 120 [66.3, 189.3] | <0.001 | <0.001 | 0.11 | <0.001 | 0.544 | 0.417 | 0.861 |
| Plasma PCT  [yg/l] | 0.10 [0.06, 0.17] | 0.16 [0.10, 0.39] | 0.08 [0.07, 0.15] | 0.14 [0.07, 0.4] | 0.019 | 0.081 | 1.000 | 0.678 | 0.133 | 1.000 | 0.588 |
| Plasma WBC  [4–10 x 10^9^/l] | 9.8 [7.3, 12.6] | 10.4 [8.4, 12.5] | 13.5 [8.1, 15.3] | 11.2 [9.14, 13.94] | 0.257 | - | - | - | - | - | - |
| CSF WBC  [4–10 x 10^9^/l] | 102 [6, 240] | 41 [8, 142] | 348 [209, 1013] | 280 [88, 665] | <0.001 | 1.000 | 0.083 | 0.074 | 0.003 | <0.001 | 1.000 |

Supplementary list 1. Diagnostic criteria for VRI of the patients from the retrospective cohort. *CRP:* protein C-reactive, *CSF:* cerebrospinal fluid, *EVD:* external ventricular drainage, *PCT*: Procalcitonin, *VRI:* Ventriculostomy related infection, *WBC:* White blood cell count.

VRI was defined by the presence of at least two of the following conditions at 2 or more days after insertion of EVD:

a) clinical signs of VRI (fever, meningeal irritability, and/or unclear neurological deterioration) not better explained by an alternative origin (e.g. increasing intra- cranial pressure, progression of hemorrhaging etc.);

b) white blood cell count (WBC) in CSF >500/μL;

c) positive microbiological CSF culture;

d) elevated systemic inflammatory parame- ters (C-reactive protein (CRP) > 5mg/l, procalcitonin (PCT) >0.1 ug/l, and or WBC > 9.6 G/l);

e) absence of alternative infectious origin (excluded by performing blood, bronchial and urinary cultures, plain chest X-rays and physical examination).
